# Survival trends and prognostic factors for patients with extramedullary plasmacytoma: A population-based study

**DOI:** 10.3389/fonc.2022.1052903

**Published:** 2022-12-13

**Authors:** Xuxing Shen, Lina Zhang, Jing Wang, Lijuan Chen, Shu Liu, Run Zhang

**Affiliations:** ^1^ Department of Hematology, Collaborative Innovation Center for Cancer Personalized Medicine, Jiangsu Province Hospital, The First Affiliated Hospital of Nanjing Medical University, Nanjing, China; ^2^ Department of Hematology, Nanjing First Hospital, Nanjing Medical University, Nanjing, China; ^3^ Department of Radiation Oncology, Jiangsu Province Hospital, The First Affiliated Hospital of Nanjing Medical University, Nanjing, China

**Keywords:** extramedullary plasmacytoma, survival trends, prognostic factor, prognostic model, nomogram

## Abstract

**Background:**

Extramedullary plasmacytoma (EMP) is a localized plasma cell neoplasm that originates from tissues other than bone. The survival trends and prognostic factors of patients with EMP in recent years remain unreported.

**Methods:**

We used the SEER databases to extract the data. Survival curves were calculated using the Kaplan-Meier method and a nomogram was created based on the Cox’s proportional hazards model.

**Results:**

A total of 1676 cases of EMP were identified. Patients in period-2 (2008-2016) show similar survival (p=0.8624) to those in period-1(1975-2007). Age, gender, race, and sites were prognostic of patient outcomes. And the use of surgery was associated with improved survival. The patients were randomly assigned to the training cohort and the validation cohort in a ratio of 2:1. Four factors including age, gender, race, and sites were identified to be independently predictive of the overall survival of patients with EMP. A prognostic model (EMP prognostic index, EMP-PI) comprising these four factors was constructed. Within the training cohort, three risk groups displayed significantly different 10-year survival rates: low-risk (73.0%, [95%CI 66.9-78.2]), intermediate-risk (39.3%, [95%CI 34.3-44.3]), and high-risk (22.6%, [95%CI 15.3-30.9]) (p<0.0001). Three risk groups were confirmed in the internal validation cohort. We also constructed a 5-factor nomogram based on multivariate logistic analyses.

**Conclusion:**

The survival of patients with EMP did not improve in recent years. The EMP-PI will facilitate the risk stratification and guide the risk-adapted therapy in patients with EMP.

## Introduction

Extramedullary plasmacytoma (EMP), which is also known as extraosseous plasmacytoma, refers to a localized plasma cell neoplasm that originates from tissues other than bone. EMP is rare, constituting only 2% of all plasma cell malignancies (1). The median age of patients of EMP is approximately 65 years, and two-thirds of patients are male (2). The most common site of EMP is the upper aerodigestive tract (UAD), however, sites including the gastrointestinal tract, lymph nodes, bladder, breasts, and others may also be involved by EMP (2). The establishment of the diagnosis of EMP requires no involvement of the bone marrow, which is usually assessed by radiological and morphological studies. And clinical features indicating plasma cell myeloma are absent in patients with EMP. The mainstay for the treatment of patients is radiation, which always results in a good response (3). However, the prognosis of patients with EMP is heterogeneous. Local relapse occurs in 14-20% of patients with EMP, and less commonly, patients also experience a relapse in distant extraosseous sites (4, 5). As compared to solitary plasmacytoma of the bone (SPB), the progression to myeloma in EMP is less common, occurring in 25%-35% of cases at 10 years (65%-84% in SPB) (6, 7).

The heterogeneity in the outcomes of patients with EMP suggests the requirement for prognostic tools as well as an unmet need in treatment. However, due to the rarity of EMP, the prognostic factors and the best therapeutic approaches remain less well defined. Some studies have investigated the prognostic factors for patients with EMP (8–11). For example, minimal bone marrow involvement was associated with an higher risk of progression to plasma cell myeloma (3). However, most of these studies are retrospective studies with small numbers of cases (9–11). Large case series studies using databases including the Surveillance, Epidemiology, and End Results (SEER) database and the National Cancer Data Base has been used to explore the clinical characteristics, survival trends, and prognostic factors in patients with EMP (2, 12–14). However, some of these studies were focused on EMP in specific anatomical sites (12, 13) while others include cases of SBP in their analysis (14). Until now, large database analysis focused on the entire cohort of patients with EMP was very limited (2). Moreover, there are no established prognostic models for predicting the survival of patients with EMP.

The use of novel drugs including bortezomib and lenalidomide has dramatically prolonged the survival of patients with plasma cell myeloma (15–18). As progression to myeloma occurs in a proportion of patients with EMP, use of the novel agents could be also effective in this setting. Therefore, we postulate that novel agents could have improved the outcomes of patients with EMP in recent years. In the current study, we studied the survival trends of patients with EMP, which could at least partly reflect the benefit provided by the novel agents. We also analyzed the prognostic impacts of baseline characteristics in patients with EMP. After the identification of significant prognostic factors, we established a prognostic model using a training cohort plus a validation cohort and a 5-factor nomogram to predict the prognosis of patients with EMP.

## Materials and methods

### Data source

We used the 18 SEER databases of the National Cancer Institute in the United States to extract data for the analysis. The SEER database represents the US population, with data in patient-level abstracted from 18 geographically diverse populations including rural, urban, and regional populations.

### Data collection

The third edition of the International Classification of Disease for Oncology (ICD-O-3) 9734 was used to identify cases of EMP. The ICD-O-3 helps us identify localized disease and myeloma with extramedullary plasmacytoma involvement or disseminated extra extramedullary were not included. So all the cases in our study were considered as “solitary extramedullary plasmacytoma”. Cases diagnosed from 1975 to 2016 were included in this study. Patients with bone marrow, peripheral blood, bone, or multiple lymph nodes involvement, bilateral involvement, unknown information of site, or survival of 0 months were excluded from this study. For each case, baseline factors including age at the time of diagnosis, gender, race, site, insurance status, and use of surgery were collected. Sites including the nose, paranasal sinuses, oral cavity, pharynx, larynx, and salivary glands were classified as upper aerodigestive tract (UAD). Other sites were classified as non-UAD.

### Construction of a predictive nomogram

A predictive nomogram was developed using Cox’s proportional hazards models. Candidate variables including sex, race,age,and the status of UAD or surgery. The area under the receiver operating curve (AUC/C-statistic) and Brier score were calculated to evaluate the performance of the model. The model was validated internally using bootstrap method with a total of 100 replications.

### Statistical analysis

As novel agents were widely used for multiple myeloma treatment after 2007 (19), we divided these patients into those diagnosed from 1975 to 2007 and those from 2008 to 2015 to study the potential benefits of novel agents in the treatment of EMP. And we compared the differences in patient demographics by using Fisher’s exact test or the chi-square test. For exploring the difference in survival, survival curves were constructed using the Kaplan-Meier method and the log-rank test was used for comparing the difference. Multivariate analysis was performed by using the multivariate Cox model. All tests were 2-sided and P<0.05 was defined as statistically significant. GraphPad Prism 8.0.1 statistical software or R version 4.1.0 was used for data analysis.

## Results

### Baseline characteristics of patients with extramedullary plasmacytoma

A total of 1676 (from 1975 to 2016) cases with EMP were included in this study. Of these cases of EMP, 837 and 839 cases were from 1975-2007 (period 1) and 2008-2016 (period 2), respectively. The median age of the patients with EMP was 63 years (IQR: 52-74 years), and two-thirds (65.8%) of the patients were male. Six hundred and seventy-two cases (40.1%) were found in the UAD and 1004 cases (59.9%) were found in other sites. Most of the patients were white (1339, 80.4%), 212 (12.7%) were black, and 114 (6.9%) were from other races. Less surgery was performed and fewer EMP cases from UAD were diagnosed in period 2 when compared with period 1. Other baseline features were summarized in **Table 1**.

**Table 1:** Baseline characteristics of patients with EMP.

Baseline characteristics	Number (%)	1975-2007 (n=837)	2008-2016 (n=839)	*P* value
***Age***				
≤65	932 (55.6%)	479 (57.2%)	453 (53.9%)	0.1826
>65	744 (44.4%)	358 (42.8%)	386 (46.1%)	
***Gender***				
Male	1103 (65.8%)	562 (67.1%)	541 (64.5%)	0.2505
Female	573 (34.2%)	275 (32.9%)	298 (35.5%)	
***UAD and non-UAD***				
UAD	672 (40.1%)	355 (42.4%)	317 (37.8%)	0.0531
Non-UAD	1004 (59.9%)	482 (57.6%)	522 (62.2%)	
***Insurance status***				
Insured	692 (83.2%)	37 (88.1%)	655 (82.9%)	0.3815
Uninsured	140 (16.8%)	5 (11.9%)	135 (17.1%)	
***Race***				
White	1339 (80.4%)	680 (82.0%)	659 (78.8%)	0.2347
Black	212 (12.7%)	95 (11.5%)	117 (14.0%)	
others	114 (6.9%)	54 (6.5%)	60 (7.2%)	
***Surgery***				
Performed	782 (47.0%)	451 (54.1%)	331 (39.9%)	**<0.0001**
Not performed	882 (53.0%)	383 (45.9%)	499 (60.1%)	
***COD***				
MM	281 (35.6%)	159 (30.0%)	122 (47.1%)	**<0.0001**
Other causes	508 (64.4%)	371 (70.0%)	137 (52.9%)	

EMP, extramedullary plasmacytoma; UAD, upper aerodigestive tract; COD, cause of death; MM, multiple myeloma. The bold values (<0.0001) means p value <0.05.

### Survival trends of patients with EMP

**Figure 1:**
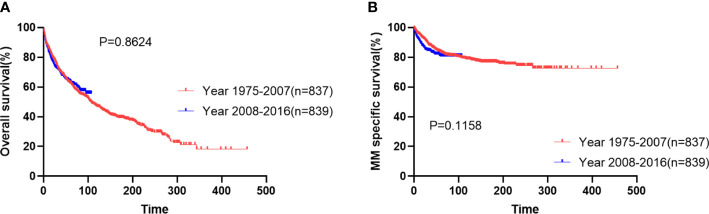
Overall survival **(A)** and myeloma-specific survival **(B)** of patients with extramedullary plasmacytoma in two periods.

The median follow-up was 45 months (IQR: 16-105 months). The median survival was 108 months and the 10-year survival rate was 47.4% (95%CI 44.4-50.3). The most common cause of death was myeloma, accounting for 35.6% of the causes of death. The median myeloma-specific survival was not reached, and the 10-year myeloma specific survival rate was 77.6% (95%CI 75.0-80.1). The overall survival of patients diagnosed in period 1 was similar to that of patients in period 2 (**Figure 1A**, median OS: 107 months vs not reached, p=0.8624). The 5-year myeloma-specific survival of patients diagnosed in period 1 was 83.2%, similar to that of patients in period 2 (80.6%, **Figure 1B**, p=0.1158). No significant improvement was observed in OS or myeloma-specific survival for patients diagnosed in the new era.

### The impacts of baseline characteristic and surgery on the outcomes of patients

**Figure 2:**
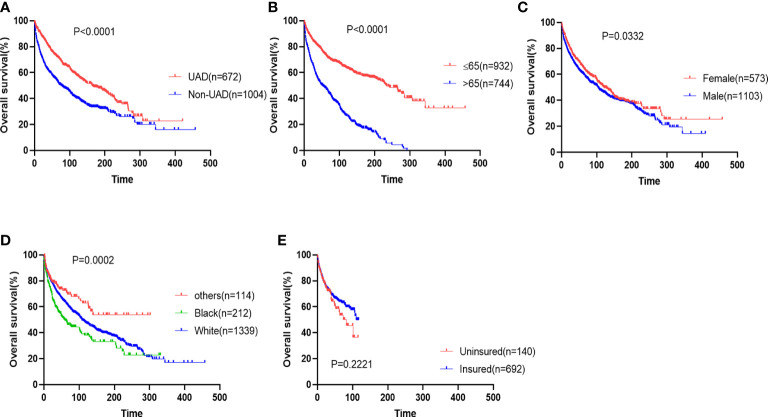
The impacts of sites **(A)**, age **(B)**, gender **(C)**, race **(D)**, and insurance status **(E)** on overall survival of patients with extramedullary plasmacytoma.

Firstly, we analyzed the survival outcomes for the cases of EMP from different sites. Patients with EMP from the non-UAD sites showed decreased OS and myeloma-specific survival (**Figure 2A** and **Supplemental Figure 1A**). The prognostic impacts of other baseline characteristics including age, gender, race, and insurance status were analyzed. We found that age (>65 *vs.* ≤65), gender (male *vs.* female), and race (black *vs.* white/others) were significant predictors of OS (**Figures 2B–E**). Additionally, we found that age and race were also significantly associated with myeloma-specific survival (**Supplemental Figures 1B–E**).

**Figure 3:**
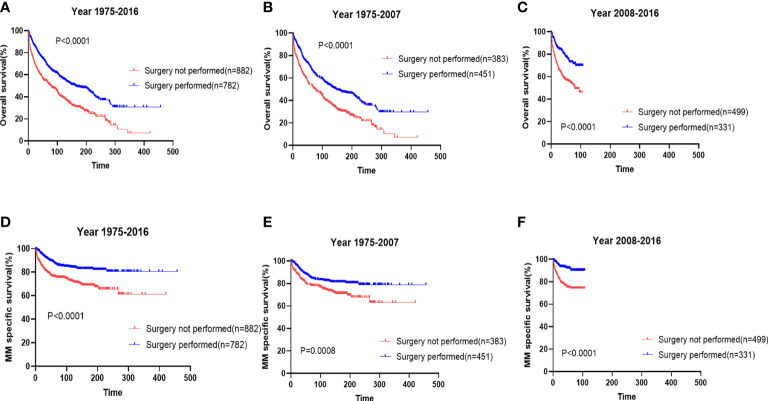
The prognostic effects of surgery on the overall survival **(A)** and myeloma-specific survival **(D)** of patients with extramedullary plasmacytoma (EMP). The prognostic effects of surgery on the overall survival and myeloma-specific survival of patients with diagnosed from 1976-2007 **(B, E)** and those diagnosed from 2008-2016 **(C, F)**.

We also analyzed the prognostic impact of the use of surgery in patients with EMP. We demonstrated that surgery remarkably improved the survival (**Figure 3A**, median: surgery 163 months *vs.* no surgery 77 months; p<0.0001) and myeloma-specific survival (**Figure 3D**, median: not reached vs. no reached; p<0.0001) of patients with EMP. The prognostic impacts of the use of surgery were maintained in two periods (**Figures 3B, C, E, F**).

### Multivariable analysis for overall survival and development of a prognostic model for patients with EMP

Multivariable analysis was then conducted to evaluate the independent prognostic impacts of age, gender, race, and sites. A total of 1665 patients with available data for all these four factors were included for further analysis. These patients were randomly assigned in a 2:1 ratio to a training cohort (n=1110) or an internal validation cohort (n=555). Multivariable analysis of prognostic factors for OS was performed in the training cohort and the internal validation cohort, respectively. And age, gender, race, and sites were identified as independent prognostic factors in both the training cohort and the internal validation cohort (**Table 2**). According to the multivariable analysis of prognostic factors for OS, all these four factors were included to construct a prognostic model for patients with EMP (EMP prognostic index; EMP-PI). The individual weighted risk scores of the independent factors were determined according to the regression analysis parameters. Therefore, weighted risk scores of 1 were assigned to gender, race, and sites, and a weighted score of 2 was assigned to age. Finally, the total risk scores (EMP-PI) ranged from 0 to 5.

**Table 2:** Multivariable analysis of prognostic factors for overall survival in training cohort and validation cohort.

group	prognostic factor	Adverse factor	HR (95% CI)	p value	Assigned risk score
Training cohort	Age	>65 years	3.0 (2.5-3.6)	<0.001	2
	Gender	Male	1.2 (1.0-1.5)	0.036	1
	Race	Black	1.4 (1.1-1.8)	0.010	1
	Sites	Non-UAD sites	1.8 (1.5-2.2)	<0.001	1
Validation cohort	Age	>65years	2.7 (2.1-3.5)	<0.001	2
	Gender	Male	1.6 (1.2-2.1)	0.001	1
	Race	Black	1.4 (1.0-2.0)	0.033	1
	Sites	Non-UAD sites	1.7 (1.3-2.1)	<0.001	1

HR, Hazard ratio; CI, confidence interval; UAD, upper aerodigestive tract.

**Figure 4:**
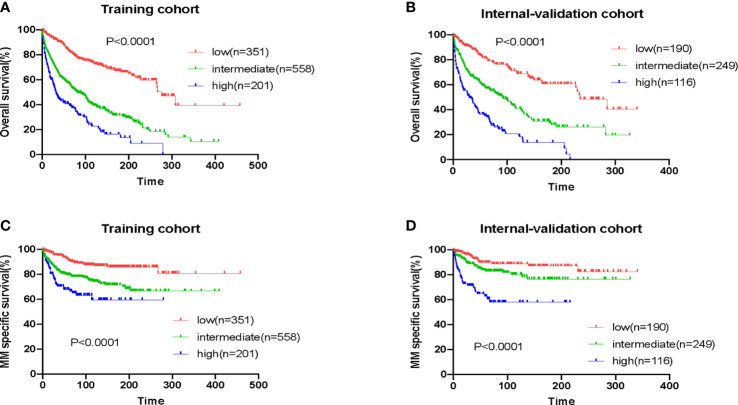
Overall survival and myeloma-specific survival according to EMP-PI: overall survival of patients with extramedullary plasmacytoma (EMP) from three risk groups in the training cohort **(A)** and the validation cohort **(B)**; myeloma-specific survival of patients with EMP from three risk groups in the training cohort **(C)** and the validation cohort **(D)**.

We stratified patients in the training cohort into three risk groups: low-risk (EMP-PI: 0-1), intermediate-risk (2, 3), and high-risk (4, 5). The 10-year survival rates for these three risk groups were significantly different (p<0.0001): for patients in the low-risk group (73.0% [95%CI 66.9-78.2]), for patients in the intermediate-risk group (39.3% [95%CI 34.3-44.3]), and for the high-risk group (22.6% [95%CI 15.3-30.9]) (**Table 3**, **Figure 4A**). Remarkably different myeloma-specific survival was also observed for these three risk categories (p<0.0001; **Supplemental Table 1**, **Figure 4C**). The discriminating power of the proposed EMP-PI for both OS (**Table 3**, **Figure 4B**; p<0.0001) and myeloma-specific survival (**Supplemental Table 1**, **Figure 4D**; p<0.0001) was fully confirmed based on the internal validation cohort.

**Table 3:** Survival data of three risk groups based on EMP-PI in the training and internal-validation cohort.

	EMP-PI risk score	No.	Median OS	5-year OS (95%CI)	10-year OS (95%CI)	comparison	HR (95%CI)
**Training group**		1110					
low	0-1	351 (31.6%)	277	83.3 (78.5-87.2)	73.0 (66.9-78.2)		
intermediate	2-3	558 (50.3%)	81	57.3 (52.7-61.6)	39.3 (34.3-44.3)	vs 0-1	3.0 (2.4-3.6)
high	4-5	201 (18.1%)	32	40.8 (33.1-48.2)	22.6 (15.3-30.9)	vs 2-3	1.6 (1.3-2.1)
**Internal-validation group**		555					
low	0-1	190 (34.2%)	235	81.3 (74.2-86.6)	68.8 (59.8-76.2)		
intermediate	2-3	249 (44.9%)	92	59.3 (52.5-65.6)	40.9 (33.3-48.2)	vs 0-1	2.5 (1.9-3.3)
high	4-5	116 (20.9%)	31	36.4 (27.1-45.7)	20.7 (12.5-30.3)	vs 2-3	2.0 (1.4-2.7)

EMP-PI, extramedullary plasmacytoma prognostic index; OS, overall survival; CI, confidence interval; HR, Hazard ratio.

### Construction and validation of the nomogram

**Figure 5:**
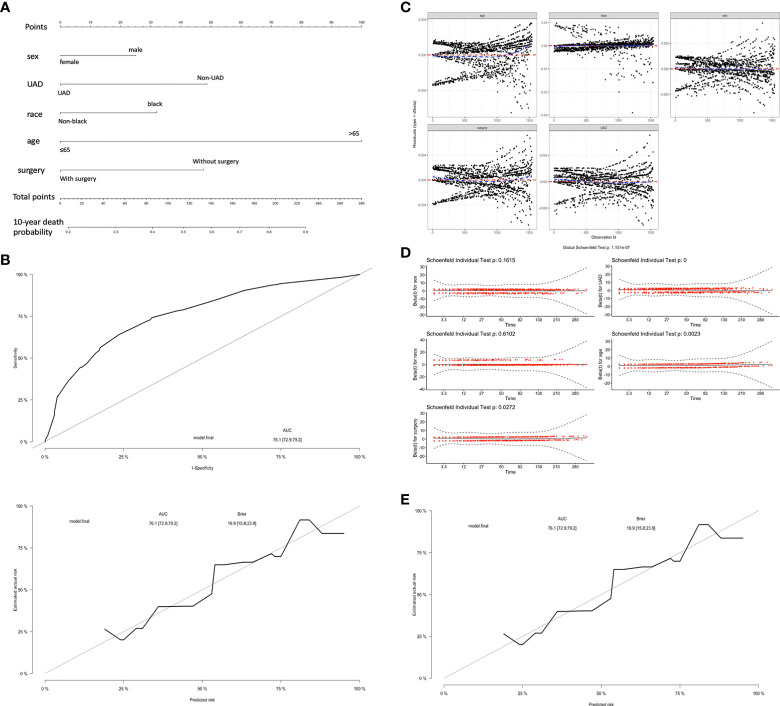
A nomogram model for 10-year death probility of patients with EMP **(A)**. The ROC plot predicted 10-year death probility for the AUC was 0.761 (95% CI 0.729–0.792) and the Brier score obtained from the model was 0.199 (95% CI 0.158–0.239) **(B)**. Outlier detection was designed by deviation residual test **(C, D)**.The value of AUC and the Brier score in the internal validation **(E)**.

We established a nomogram including sex, race, age, the status of UAD, and surgery to predict 10-year death probility (**Figure 5A**). The ROC plot showed a good performance of this nomogram in predicting 10-year death probility for the AUC was 0.761 (95% CI 0.729–0.792) (**Figure 5B**). The Brier score obtained from the model was 0.199 (95% CI 0.158–0.239) which confirmed that the calibration of this model was acceptable. No outliers were found by the bias residual test and the Schoenfeld residuals indicated that the Cox model was consistent with the proportional hazards hypothesis (**Figures 5C, D**).For the intel validation, the AUC was 0.722 (95% CI 0.688–0.758) and the Brier score was 0.195 (95% CI 0.183–0.211), respectively (**Figure 5E**).

## Discussion

In this study, we analyzed the baseline characteristics and survival of patients of EMP. We found the survival of patients with EMP has not been improved in the new era. We also found that factors including age, gender, race, and sites were independently associated with survival. By integrating these factors, we constructed a prognostic tool EMP-PI and categorized patients from the training cohort into three risk groups. Within the training cohort, the OS and myeloma-specific survival of patients with EMP were significantly different among these three groups, suggesting EMP-PI was robust in predicting the prognosis in patients with EMP. The prognostic role of EMP-PI was also confirmed by using an internal validation cohort.

The survival of patients with myeloma has improved significantly due to the use of novel drugs including bortezomib, lenalidomide, and others. We postulated that patients with EMP progressing to myeloma might benefit from the use of novel agents (20, 21). Therefore, it was possible the OS and myeloma-specific survival of patients could improve in the new era. However, we found the OS and myeloma-specific survival of patients with EMP did not improve in the new era. This observation could be attributable to the low proportion of patients with EMP who progress to myeloma. In a population-based study, with a median follow-up of 89 months, only 12% of patients with EMP developed multiple myeloma, while 70% of patients with SPB developed multiple myeloma (6). Therefore, even if the survival patients with EMP progressing to myeloma could improve in the new era, the improvement in the survival of the small proportion of patients may not be translated into the improvement in OS of the entire population of patients with EMP. Other reasons for this observation remain unknown.

Age, which is a universal prognostic factor in patients with cancer, significantly predicted the survival of patients with EMP in the study. In addition to age, the other three independent prognostic factors were identified. Being female was associated with better survival. This finding was in contrast to that in patients with SPB, in which male patients had better survival than female patients (22), further supporting that EMP and SPB are distinct entities with different clinical and biological characteristics. We also found that African American patients with EMP had the significantly worse OS and MSS than others. The differences in biological features and access to medical care may partly account for this phenomenon (23). In consistent with a previous study (13), we found that cases of EMP occurring at the UAD carried significantly better survival than those from other sites. EMPs from different sites may have different biological characteristics, which contribute to different clinical behaviors and response to therapy.

Radiation remains to be the major treatment for patients with EMP (24). In our study, surgery was significantly associated with improved OS and myeloma-specific survival. The reason accouting for the improvement in survival related to the use of surgery is not well defined. A possible explanation is that surgery debulks the tumor burden and removes the clones that could become resistant to the radiation therapy, thereby contributing to the improved survival.

Ghiassi-Nejad et al. have studied the survival trends of patients with EMP using a different database-the National Cancer Data Base (2). In consistence with our study, their study found that African American patients with EMP had worse survival. Additionally, this study showed that EMPs occurring at the head and neck region carried significantly better survival than EMPs at other sites. This finding is consistent with ours, as cases of EMP from the UAD and cases of EMP from the head and neck region are almost the same (13). However, in their study, the prognostic role of gender was not observed and the role of surgery was not studied. In the study by Gerry et al (13), in patients with EMP in the head and neck region, patients treated with surgery alone or a combination of surgery and radiation had superior survival than those treated with radiation alone; and in patients with EMP from other sites, those receiving surgery alone had superior OS but not disease-specific survival. And in another study, patients with UAD EMP treated with a combination of surgery and radiation had better OS than those treated with surgery or radiation alone, suggesting adding surgery to radiation may improve outcomes of patients with UAD EMP (25). The findings of these studies, along with that of ours, suggest patients with EMP may benefit from the use of surgery.

The strength of our study is that we developed a prognostic model that stratifies our patients into three risk groups. And the EMP-PI remained robust in predicting the survival outcomes in the validation cohort. The 10-year survival rate of patients in the low-risk group was approximately 70%, while the 10-year survival rate of those in the high-risk group was approximately 20%. It suggests that although the prognosis of the entire cohort of patients of EMP is good, patients in the high-risk group have a poor prognosis. Different treatment strategies are warranted to improve the outcomes of EMP patients in the high-risk group. This prognostic index may facilitate the risk stratification and risk-adapted therapy in patients with EMP. Finally, we constructed a nomogram by integrating five factors, and this nomogram was demonstrated to be robust in predicting the survival of patients with EMP.

Our study has some limitations. The retrospective nature of the SEER database should be admitted. As we know, advanced imaging is very important for the accurate diagnosis and staging of plasma cell dyscrasias (26), however, the imaging data are not provided by the SEER database. Additionally, the important prognostic factors including the size of the tumor, genetic aberrations, and laboratory values (lactate dehydrogenase and others) are not available in the SEER data. Further, the details regarding the use of surgery are currently not provided. A prospective study may be helpful to solve these problems.

## Conclusion

In conclusion, the survival of patients with EMP has not been improved in the new era. We identified several potential prognostic factors and developed a prognostic index that was robust in predicting the outcomes of patients. As the molecular characteristics of EMP remain unknown, more studies are needed to investigate the genetic aberrations and the mechanisms underlying the pathogenesis of EMP. Further, studies exploring novel treatment strategies are warranted to improve patients with EMP, especially those in the high-risk group.

## Data availability statement

Publicly available datasets were analyzed in this study. This data can be found here: SEER database.

## Author contributions

XS, LZ, and SL designed and wrote the manuscript. JW organized the data materials. LC and RZ supervised the study. All authors contributed to the interpretation of the data and revised the manuscript critically.
