# Supplementary material for: Survival trends and prognostic factors for patients with extramedullary plasmacytoma: A population-based study
Source: Front Oncol. 2022 Dec 13;12:1052903. doi: 10.3389/fonc.2022.1052903 (PMC9792764; doi:10.3389/fonc.2022.1052903)
Supplement: Supplementary file 1 [file DataSheet_1.pdf]

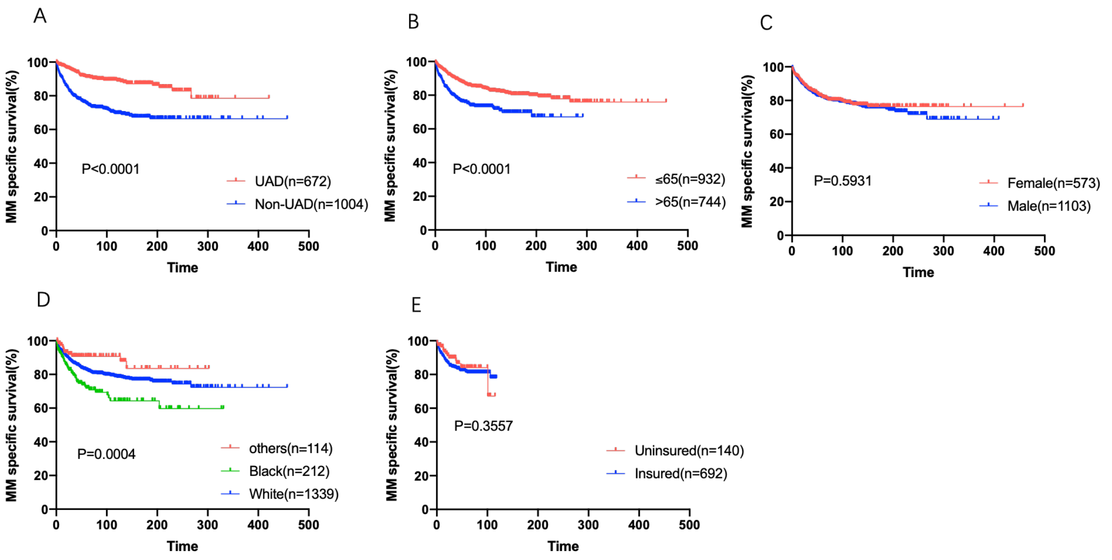

Supplemental Figure1. The impacts of sites (A), age (B), gender (C), race (D), and insurance status (E) on MM-specific survival of patients with extramedullary plasmacytoma. MM, multiple myeloma.

Supplemental Table1. Survival data of three risk groups based on EMP-PI in the training and internal-validation cohort.

	risk score	No.	Median CSS	5-year CSS (95%CI)	10-year CSS (95%CI)	vs	Hazard ratio (95%CI)
**Training group**		1110					
low	0-1	351 (31.6%)	NR	90.7 (86.6-93.6)	86.8 (81.6-90.5)		
intermediate	2-3	558 (50.3%)	NR	79.5 (75.3-83.0)	74.1 (68.8-78.6)	*vs* 0-1	2.5 (1.8-3.5)
high	4-5	201 (18.1%)	NR	67.9 (58.8-75.4)	59.4 (47.3-69.6)	*vs* 2-3	1.6 (1.1-2.4)
**Internal-validation group**		555					
low	0-1	190 (34.2%)	NR	89.5 (83.4-93.5)	88.5 (82.1-92.8)		
intermediate	2-3	249 (44.9%)	NR	82.8 (76.3-87.6)	80.2 (72.7-85.8)	*vs* 0-1	1.8 (1.1-3.1)
high	4-5	116 (20.9%)	NR	62.5 (50.7-72.3)	58.0 (45.9-68.7)	*vs* 2-3	2.7 (1.6-4.5)

HR: hazard ratio; CI: confidence interval; CSS: cause specific survival
